# Interactive correlations between artificial light at night, health risk behaviors, and cardiovascular health among patients with diabetes: A cross‐sectional study

**DOI:** 10.1111/1753-0407.70008

**Published:** 2024-10-13

**Authors:** Yi Zhang, Keyan Hu, Ying Tang, Qing Feng, Tian Jiang, Liwen Chen, Xin Chen, Chunhan Shan, Chen Han, Wenhui Chu, Nanzhen Ma, Honglin Hu, Hui Gao, Qiu Zhang

**Affiliations:** ^1^ Department of Endocrinology The First Affiliated Hospital of Anhui Medical University Hefei China; ^2^ Department of Maternal, Child and Adolescent Health, School of Public Health Anhui Medical University Hefei China; ^3^ The First Affiliated Hospital, and College of Clinical Medicine of Henan University of Science and Technology Luoyang China; ^4^ School of Nursing Anhui Medical University Hefei China; ^5^ Department of Pediatrics The First Affiliated Hospital of Anhui Medical University Hefei China; ^6^ Hospital of Anhui Medical University Hefei China

**Keywords:** cardiovascular disease, cardiovascular health, diabetes, health risk behavior, outdoor light at night

## Abstract

**Background:**

Artificial light at night (ALAN) is a common phenomenon and contributes to the severe light pollution suffered by more than 80% of the world's population. This study aimed to evaluate the relationship between outdoor ALAN exposure and cardiovascular health (CVH) in patients with diabetes and the influence of various modifiable factors.

**Methods:**

A survey method based on the China Diabetes and Risk Factor Monitoring System was adopted. Study data were extracted for 1765 individuals with diabetes in Anhui Province. Outdoor ALAN exposure (nW/cm^2^/sr) within 1000 m of each participant's residential address was obtained from satellite imagery data, with a resolution of ~1000 m. Health risk behaviors (HRBs) were measured via a standardized questionnaire. A linear regression model was employed to estimate the relationship between outdoor ALAN, HRBs, and CVH.

**Results:**

Participants' mean age was 59.10 ± 10.0 years. An association was observed between ALAN and CVH in patients with diabetes (*β* = 0.205) and exercise (*β* = −1.557), moderated by HRBs, or metabolic metrics. There was an association between ALAN, ALAN, vegetable intake, and CVH.

**Conclusions:**

Exploring the relationship between ALAN exposure and cardiovascular and metabolic health provides policy data for improving light pollution strategies and reducing the risk of cardiovascular and metabolic disease in patients with diabetes.

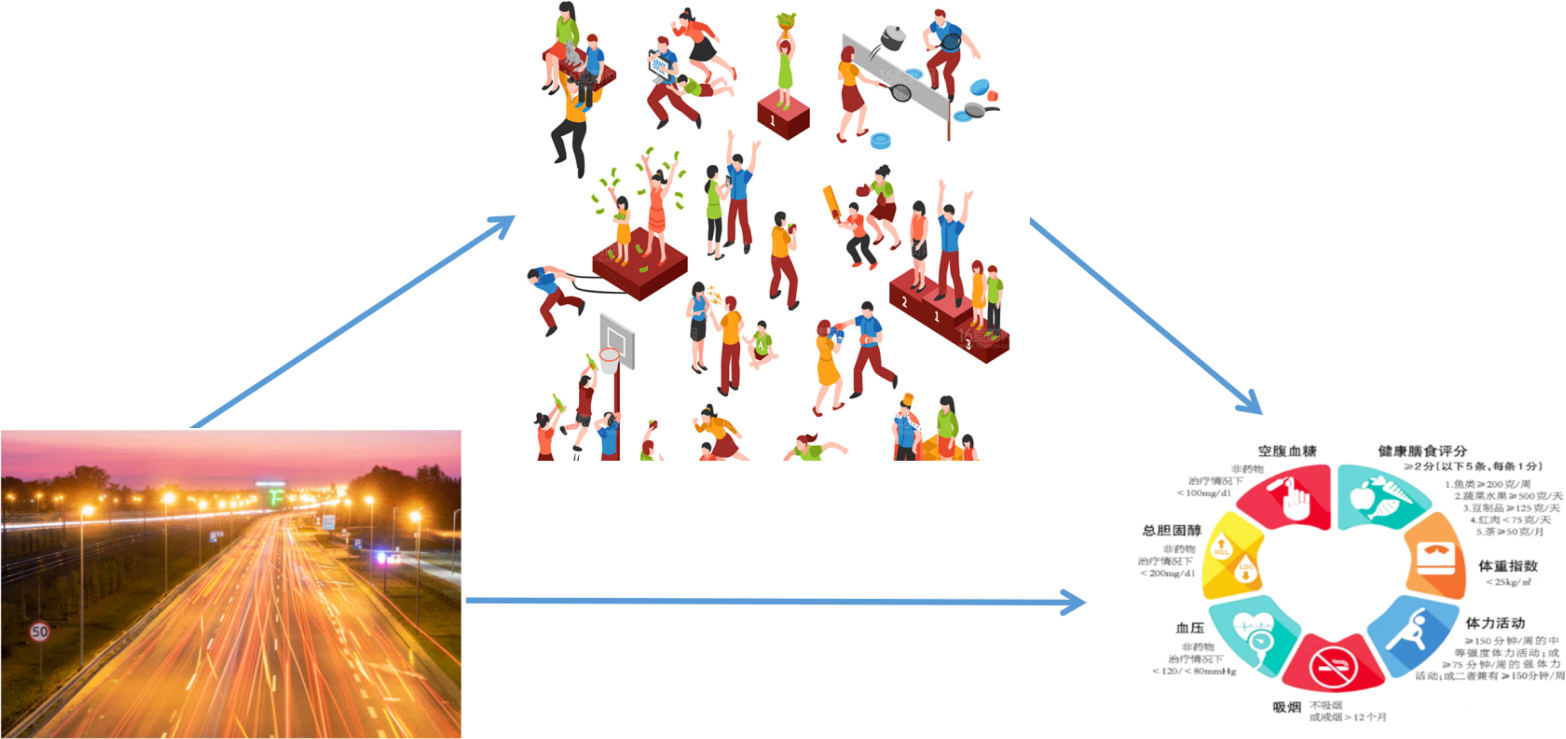

## Graphical abstract

- This study can help solve and expand public health problems by exploring the environmental factors of diabetes combined with cardiovascular disease and moving the problem forward.
- Artificial light at night (ALAN) is not only related to cardiovascular health but also plays a role in the development of cardiovascular disease together with other factors.
- By focusing on ALAN and green space at the same time, potential correlations between complementary factors can be discovered.

## 1 INTRODUCTION

As the global standard of living continues to improve, the adverse consequences of noncommunicable diseases (NCDs), such as cardiovascular disease (CVD), hypertension, and diabetes, has become a growing concern.^1^ Notably, diabetes is a significant risk factor and a leading cause of death among individuals with CVD.^2^ Consequently, monitoring the risk factors, including cardiovascular and metabolic health indicators, is particularly important as they can help predict CVD progression. Additionally, obesity, diet, physical activity levels, and smoking are modifiable factors that can contribute to the development of type 2 diabetes (T2DM) and CVD. A novel method to predict CVD has been proposed, centering on the establishment of cardiovascular health (CVH),^3^ which is particularly important in CVD development^4^ in the context of underlying diseases, ultimately underscoring the importance of exploring its causes.

Researchers have made significant contributions to identifying key metabolic and modifiable behavioral risk factors associated with diabetes and CVD. However, despite the discovery and implementation of related mitigation measures, the incidence has continued to increase and mortality has not decreased. Disturbances in the circadian rhythm are closely related to key CVH indicators. Environmental and behavioral factors, including light exposure, can cause such disruptions. Recently, the link between such disturbances, diabetes mellitus, and CVD has become increasingly clear.^5^ However, the impact of artificial light at night (ALAN) and CVH on patients with diabetes remains underexplored; moreover, questions regarding the potential factors involved and the underlying mechanisms remain unanswered. Furthermore, this topic has sparked considerable debate. From a practical viewpoint, social and economic development is intrinsically tied to outdoor artificial lighting. While ALAN reflects the progress of urban development, excessive artificial lighting also contributes to light pollution, which causes environmental challenges. Indeed, varying degrees of ALAN exposure and at different times has become a widespread issue, especially in industrialized countries.^6^, ^7^, ^8^ Shift work is a characteristic manifestation of this phenomenon.^6^ Numerous studies discuss the effects of shift work and exposure to ALAN on human health. Given that light and dark exposure patterns and normal circadian rhythms play key roles in many behavioral and physiological functions,^6^, ^9^ working and exposure to light at night have been deemed detrimental to human health and well‐being to varying degrees.^6^, ^10^, ^11^, ^12^, ^13^, ^14^, ^15^ ALAN has been associated with biomarkers of circadian rhythm disturbances and adverse health outcomes, which prompts further investigation into why and how it may contribute to disease,^16^ especially in patients with diabetes.

Light pollution may play an important role in human health and mood and has direct or indirect effects.^17^ Even when dietary intake and physical activity levels are consistent, long‐term exposure to ALAN can increase body weight and disrupt glucose production and change.^18^ The disruption of circadian rhythms caused by light exposure increases metabolic abnormalities and the risk of obesity and diabetes. As a nationally representative study spanning 162 study sites, the 2010 China Chronic Noncommunicable Disease Surveillance Study included 98 658 participants over 18 years of age who had resided at the current study site for at least 6 months. Outdoor ALAN exposure level was positively correlated with HBA1c, fasting, and 2‐h blood glucose concentration, homeostasis model assessment of insulin resistance (HOMA‐IR), and negatively correlated with β cell function HOMA (HOMA‐B), which also provides direct evidence that ALAN causes abnormal glucose and lipid metabolism. In addition, long‐term exposure to high‐intensity outdoor ALAN was associated with an increased risk of impaired glucose homeostasis and diabetes prevalence.^19^ Other studies have established a statistically significant relationship between outdoor ALAN exposure and weight gain and cardiovascular problems.^20^ A prospective cohort study of older adults in Hong Kong, China, identified an expose‐response relationship between nighttime light exposure and hospitalization and mortality from coronary heart disease. Studies have also reported an association between ALAN and metabolic syndrome (MetS), and a stratified analysis showed that this association was stronger in men, older adults, urban residents, people who engage in less physical activity, and people living in areas with lower green vegetation coverage.^21^

Environmental factors are being increasingly recognized as important contributors to the treatment of chronic diseases and provide a good basis for healthcare workers to specify interventions.^22^ Empirical research suggests that nature exposure (especially green space) and physical activity may serve as an important mechanistic pathway to beneficial health outcomes.^23^ An exposome approach review demonstrated that both external and internal exposome had an increased risk of T2DM. The review also verified that lifestyle factors such as physical activity or diet were correlated with T2DM.^24^ Liu et al. confirmed the relationship between the normalized difference vegetative index (NDVI) and five CVD events. Their study additionally provides robust evidence for the beneficial effects of green space exposure on CVH.^25^

Although current research suggests that ALAN is a significant factor in NCDs, the specific psychosocial and biochemical factors involved have not been fully characterized. Hence, focused research is needed among patients with diabetes as a more specific group. Current research indicates that the treatment of diabetes remains inadequate, thus requiring a multilevel, systematic, and comprehensive program for screening and prevention. Accordingly, any platform should encompass the treatment of diabetes and its complications, thereby establishing a solid foundation for the development of relevant guidelines or health prevention techniques. Meanwhile, few relevant studies on this subject exist, including within China.

Accordingly, we investigate the prevalence of CVH among patients with diabetes and the influence of potential ALAN‐related factors. In particular, we investigate the interactive correlations between ALAN, behavior, socioeconomic status (SES) or gender, NDVI, and CVH. Additionally, based on a sample of Chinese patients with diabetes, we assess the moderating effect of behavior and SES (or gender) on the relationships between ALAN and CVH.

## 2 METHODS

### 2.1 Survey participants

This is a cross‐sectional study, which was based on the National Diabetes and Complications Surveillance Program. The analysis explores the relationship between ALAN and health risk behaviors (HRBs) and the prevalence of diabetes and its complications.

Our research methods and contents were conducted in accordance with relevant guidelines and regulations, and the informed consent of all participants was obtained. The study was carried out in accordance with the guidelines in the Declaration of Helsinki. All procedures involving participants were approved by the Ethics Committee of the First Affiliated Hospital of Anhui Medical University.

### 2.2 Sampling setting

In 2018, a nationwide screening for chronic complications of diabetes was implemented in 31 provinces/autonomous regions/municipalities, and the detailed sampling methods of the project were outlined before.^22^ Participants included people 18 years of age or older who had been diagnosed with diabetes and had lived in the survey area for more than 6 months in the 12 months prior to the survey. Pregnant women and individuals with mental illnesses were excluded.

### 2.3 Sample size estimation

The detailed sampling methods are derived from a previous study.^26^ Based on previous research findings, the prevalence of diabetes was 11.2%. Based on Equation (1), the minimum sample size was calculated to be 690 people, with a relative precision of 15% (*ɛ*), *α* = 0.05, and *Z*_1‐*α*/2_ = 1.96:

(1) $$n=\frac{\left(1-p\right)\;Z_{1-\alpha /2}}{{ɛ}^{2}p}$$

### 2.4 Investigation contents and methods

Data were obtained via a questionnaire survey, body measurements, and laboratory testing. Sampling methods are described in detail in a previous study.^26^

### 2.5 Outdoor light at night data

Outdoor ALAN data were obtained as per the protocol described in our previous study.^27^ We utilized the highest quality global nighttime light map available, which is produced by the Earth Observation Group (https://eogdata.mines.edu/).^28^, ^29^

### 2.6 CVH

CVH included central obesity, decreased high‐density lipoprotein cholesterol (HDL‐C), and elevated triglyceride (TG), blood pressure, and blood glucose, among others. Instances of two or more risk factors were designated “risk factor clusters.”^30^

### 2.7 Statistical analysis

The SPSS 23.0 statistical software was used for analysis. Mean and standard deviation (SD) were used to describe continuous variables, and their association with outcomes was analyzed using univariate analysis of variance and *T*‐test. In addition, frequency and percentage were used to describe categorical variables, and their association with outcomes were analyzed via chi‐2 tests. Multivariate logistic regression was performed to analyze the correlation between HRBs and CVH, body mass index (BMI), waist circumference (WC), other metabolic indices (e.g., albumin [Alb], TG), and NDVI.

The relationship between ALAN and CVH was examined using several models: Model 1 evaluated the relationship between general demographic and CVH. Model 2 assessed the relationships between different HRBs, such as physical activity (moderate), sleep duration, metabolic markers, and CVH. Model 3 calculated complex metabolic measures, namely, clustered metabolic risk score (zMS), to summarize standardized values of WC, fasting TG, glycosylated hemoglobin (HbA1c), reciprocal systolic blood pressure, and HDL‐C. These variables are standardized by subtracting the sample mean from the individual mean and then dividing by the standard deviation to get the corresponding value.^31^ The triglyceride glucose (TyG) index, which refers to a product of fasting blood glucose and triglycerides, was first introduced as a reliable alternative marker for insulin resistance.^32^ However, more recently, a close relationship has been established between insulin resistance and obesity, TyG‐related parameters (including TyG), and anthropometric indicators of obesity (such as TyG‐BMI and TyG‐WC), which makes them superior to insulin resistance alone in predicting insulin resistance.^33^ VAI: Male: [=(WC/[39.68 + 1.88 × BMI]) × (TG/1.03) × (1.31/HDL); Female: (WC/[36.58 + 1.89 × BMI]) × (TG/0.81) × (1.52/HDL)], where both TG and HDL levels were expressed in mmol/L.^34^

The first step of our analysis involved descriptive statistics focusing on sociodemographic factors and CVH. Second, multilevel logistic regression was adopted to evaluate the association between ALAN, HRBs, and CVH. Third, an audit analysis was conducted using established procedures,^35^ which was used to investigate the regulatory effect of ALAN, HRBs, and CVH. We also analyzed the relationship between ALAN and different types of HRBs, as well as that between ALAN, metabolic markers, and CVH. SPSS automatically calculates the interaction effect while generating the proportion of variance explained by the moderating effect of CVH on the relationship (evidenced by an increase in *r* ^2^). Our model also accounted for sociodemographic correlations, and their effects were adjusted in the moderation analysis.

## 3 RESULTS

### 3.1 General demographic statistics

The general distribution of the variables examined among CVH is presented in Table 1. Among the 1765 participants for whom questionnaire responses were obtained, 906 (51.1%) were men, the average age was 59.10 ± 10.0 years, 853 (48.1%) lived in rural areas, 548 (30.9%) lived in towns, and 371 (20.9%) lived in cities. Among all assessed demographic details, sex, educational level, income level, and SES were significantly associated with CVH. By contrast, marital status, ethnicity, and age were not correlated with CVH.

**TABLE 1:** Multilevel linear regression between independent variables and CVH.

Variable	CVH						
	*R* ^2^	*β*	*t*	*p*	*F*	LLCI	ULCI
Sex	0.046	0.870	9.141	<0.01	83.561	0.683	1.057
Age	0.00	−0.001	−0.275	>0.05	0.076	−0.011	0.008
Ethnicity	0.001	0.492	1.072	>0.05	1.150	−0.408	1.391
Marital status	0.00	−0.133	−0.349	>0.05	0.122	−0.882	0.616
Educational level	0.004	0.118	2.705	<0.01	7.317	0.032	0.204
Income level	0.009	0.195	3.993	<0.01	15.945	0.099	0.290
SES	0.026	0.160	0.392	<0.01	46.216	0.279	0.505

Abbreviations: CVH, cardiovascular health; LLCI, lower‐level confidence interval; SES, socioeconomic status; ULCI, upper‐level confidence interval.

### 3.2 Association between independent variables and CVH

The multilevel linear regression results and association with CVH are presented in Table 2. A dose–response relationship was observed between different ALAN and CVH (*β* = 0.205). After controlling for covariates, these relationships remained significant. A significant relationship between exercise (*β* = −1.557), vegetable intake (*β* = −1.022), walking (*β* = −0.449), and CVH was also established. Other results are reported in Table 2.

**TABLE 2:** Multilevel linear regression between independent variables and CVH.

Variable	CVH						
	*R* ^2^	*β*	*t*	*p*	*F*	LLCI	ULCI
ALAN							
Model 1	0.042	0.205	8.786	<0.01	77.195	0.028	0.044
Model 2	0.109	0.06	6.047	<0.01	23.594	0.018	0.035
Exercise							
Model 1	0.099	−1.557	−13.909	<0.01	193.473	−1.777	−1.338
Model 2	0.176	0.924	13.523	<0.01	41.454	0.790	1.058
Vegetable intake							
Model 1	0.063	−1.022	−10.835	<0.01	117.398	−1.207	−0.837
Model 2	0.103	0.362	5.070	<0.01	22.267	0.222	0.502
Walking							
Model 1	0.012	−0.449	−4.603	<0.01	21.190	−0.640	−0.258
Model 2	0.096	−0.338	−3.548	<0.01	20.666	−0.526	−0.151
Sleep duration							
Model 1	0.07	−0.099	−3.572	<0.01	12.756	−0.154	−0.045
Model 2	0.094	−0.078	−2.893	<0.01	20.151	−0.130	−0.025
Sleep disorder							
Model 1	0.005	0.116	3.062	<0.01	9.377	0.042	0.191
Model 2	0.097	0.136	3.621	<0.01	20.730	0062	0.210
SES							
Model 1	0.026	0.160	0.392	<0.01	46.216	0.279	0.505
Model 2	0.092	0.223	2.224	<0.01	19.733	0.026	0.419

*Note*: Model 1: crude model; Model 2: controlled for parent educational level, gender, economic level, whether only child, friend numbers, residential areas, and age.

Abbreviations: ALAN, artificial light at night; CVH, cardiovascular health; LLCI, lower‐level confidence interval; SES, socioeconomic status; ULCI, upper‐level confidence interval.

### 3.3 Moderation analysis between ALAN, exercise, and behaviors on CVH

Moderation analyses were performed for ALAN, exercise, behaviors, and CVH. The results are presented in Tables 3 and 4. We identified a positive association between exercise, vegetable intake, and CVH, with vegetable intake playing a moderating role in the increased CVH risk induced by ALAN (Table 3). Similar results were derived regarding ALAN, exercise, walking, and CVH (Table 4).

**TABLE 3:** Moderation analysis between ALAN, exercise, vegetable intake, and CVH.

Variable	CVH					
	Coeff	SE	*t*	*p*	LLCI	ULCI
ALAN	0.0447	0.030	1.4913	>0.05	−0.0141	0.1036
Exercise	−1.9992	0.2150	−9.2991	<0.01	−2.4209	−1.5776
Vegetable intake	0.2778	0.0923	3.0103	<0.01	0.0968	0.4588
Int_1	−0.0158	0.0157	−1.0085	>0.05	−0.0466	0.0149
Int_2	−0.0113	0.0047	−2.4049	<0.05	−0.0204	−0.0021
Int_3	−0.1196	0.0482	−2.4797	<0.05	−0.2141	−0.0250
Int_4	0.0066	0.0025	2.6437	<0.01	0.0017	0.0115

*Note*: Int 1: ALAN × exercise; Int 2: ALAN × vegetable; Int 3: exercise × vegetable; Int 4: ALAN × exercise × vegetable.

Abbreviations: ALAN, artificial light at night; CVH, cardiovascular health; LLCI, lower‐level confidence interval; SE, standard error; ULCI, upper‐level confidence interval.

**TABLE 4:** Moderation analysis between ALAN, exercise, walking, and CVH.

Variable	CVH					
	Coeff	SE	*t*	*p*	LLCI	ULCI
ALAN	0.0529	0.0137	3.8576	<0.01	0.0260	0.0798
Exercise	1.4344	0.2800	5.1235	<0.01	0.8853	1.9835
Walking	0.0006	0.1172	0.0054	>0.05	−0.2293	0.2306
Int_1	−0.0258	0.0179	−1.4382	>0.05	−0.0609	0.0094
Int_2	−0.0204	0.0095	−2.1540	<0.05	−0.0389	−0.0018
Int_3	−0.4257	0.1828	−2.3286	<0.05	−0.7843	−0.0672
Int_4	0.0234	0.0129	1.8161	0.0695	−0.0019	0.0487

*Note*: Int 1: ALAN × exercise; Int 2: ALAN × walking; Int 3: exercise × walking; Int 4: ALAN × exercise × walking.

Abbreviations; ALAN, artificial light at night; CVH, cardiovascular health; LLCI, lower‐level confidence interval; SE, standard error; ULCI, upper‐level confidence interval.

### 3.4 Moderation analysis between ALAN, exercise, and demographic information on CVH

Moderation analyses were performed for ALAN, exercise, demographic information, and CVH. Results suggest a positive association between ALAN exposure and CVH, with exercise and sex playing a moderating role in the increased risk of CVH induced by ALAN (Table 5). We observed similar results for ALAN, exercise, age, and CVH (Table 6), ALAN, exercise, SES, and CVH (Table 7), and ALAN, exercise, residential, and CVH (Table 8).

**TABLE 5:** Moderation analysis between ALAN, exercise, sex, and CVH.

Variable	CVH					
	Coeff	SE	*t*	*p*	LLCI	ULCI
ALAN	0.1480	0.0537	2.7572	<0.01	0.0427	0.2534
Exercise	−0.9755	0.4941	−1.9743	<0.05	−1.9446	−0.0064
Sex	2.9563	0.6061	4.8774	0.0744	1.7675	4.1450
Int_1	−0.0527	0.0291	−1.8069	0.0710	−0.1098	0.0045
Int_2	−0.0956	0.0330	−2.8992	<0.01	−0.1603	−0.0309
Int_3	−1.0086	0.3177	−3.1751	<0.01	−1.6317	−0.3856
Int_4	0.0412	0.0180	2.2924	<0.05	0.0059	0.0764

*Note*: Int 1: ALAN × exercise; Int 2: ALAN × gender; Int 3: exercise × gender; Int 4: ALAN × exercise × gender.

Abbreviations: ALAN, artificial light at night; CVH, cardiovascular health; LLCI, lower‐level confidence interval; SE, standard error; ULCI, upper‐level confidence interval.

**TABLE 6:** Moderation analysis between ALAN, exercise, age, and CVH.

Variable	CVH					
	Coeff	SE	*t*	*p*	LLCI	ULCI
ALAN	0.0085	0.0144	0.5932	>0.05	−0.0197	0.0367
Exercise	0.3708	0.2706	1.3702	>0.05	−0.1600	0.9015
Age	0.0206	0.1186	0.1734	>0.05	−0.2121	0.2533
Int_1	0.0456	0.0192	2.3701	<0.05	0.0079	0.0833
Int_2	0.0110	0.0091	1.2135	>0.05	−0.0068	0.0288
Int_3	0.3312	0.1887	1.7548	>0.05	−0.0390	0.7013
Int_4	−0.0253	0.0120	−2.1081	<0.05	−0.0488	−0.0018

*Note*: Int 1: ALAN × exercise; Int 2: ALAN × age; Int 3: exercise × age; Int 4: ALAN × exercise × age.

Abbreviations: ALAN, artificial light at night; CVH, cardiovascular health; SE, standard error; LLCI, lower‐level confidence interval; ULCI, upper‐level confidence interval.

**TABLE 7:** Moderation analysis between ALAN, exercise, SES, and CVH.

Variable	CVH					
	Coeff	SE	*t*	*p*	LLCI	ULCI
ALAN	0.0098	0.0139	0.4829	>0.05	−0.0176	0.0371
Exercise	0.3048	0.2141	1.4235	>0.05	−0.1152	0.7247
SES	0.1036	0.0695	1.4898	>0.05	−0.0328	0.2399
Int_1	0.0689	0.0212	3.2564	<0.01	0.0274	0.1104
Int_2	0.0063	0.0064	0.9853	>0.05	−0.0062	0.0187
Int_3	0.2793	0.1099	2.5411	<0.05	0.0637	0.4949
Int_4	−0.0283	0.0091	−3.1158	<0.01	−0.0461	−0.0105

*Note*: Int 1: ALAN × exercise; Int 2: ALAN × SES; Int 3: exercise × SES; Int 4: ALAN × exercise × SES.

Abbreviations: ALAN, artificial light at night; CVH, cardiovascular health; LLCI, lower‐level confidence interval; SE, standard error; SES, socioeconomic status; ULCI, upper‐level confidence interval.

**TABLE 8:** Moderation analysis between ALAN, exercise, residential area, and CVH.

Variable	CVH					
	Coeff	SE	*t*	*p*	LLCI	ULCI
ALAN	−0.0945	0.0394	−2.4020	<0.05	−0.1717	−0.0173
Exercise	−3.3927	0.5406	−6.2759	<0.01	−4.4530	−2.3324
Residential area	−1.2714	0.4107	−3.0959	<0.01	−2.0768	−0.4659
Int_1	0.0515	0.0218	2.3614	<0.05	0.0087	0.0942
Int_2	0.0635	0.0227	2.7991	<0.01	0.0190	0.1080
Int_3	0.5340	0.2210	2.4166	<0.05	0.1006	0.9674
Int_4	−0.0284	0.0122	−2.3387	<0.05	−0.0523	−0.0046

*Note*: Int 1: ALAN × exercise; Int 2: ALAN × residential; Int 3: exercise × residential; Int 4: ALAN × exercise × residential.

Abbreviations: ALAN, artificial light at night; CVH, cardiovascular health; LLCI, lower‐level confidence interval; SE, standard error; ULCI, upper‐level confidence interval.

### 3.5 Moderation analysis between ALAN, exercise, and metabolic metrics on CVH

Moderation analyses were performed for ALAN, exercise, metabolic metrics, and CVH. Results suggest a positive association between ALAN exposure and CVH, with exercise and TG levels exhibiting moderating roles in the increased CVH risk induced by ALAN (Table 9). We obtained similar results for ALAN, exercise, TyG (Table 10), TG:HDL (Table 11), albumin (Alb; Table 12), VSI (Table 13), and CVH, as well as between ALAN, exercise, TyG‐BMI index, and CVH (Table 14).

**TABLE 9:** Moderation analysis between ALAN, exercise, TG, and CVH.

Variable	CVH					
	Coeff	SE	*t*	*p*	LLCI	ULCI
ALAN	0.0301	0.0059	5.1354	<0.01	0.0186	0.0416
Exercise	0.5873	0.1196	4.9120	<0.01	0.3528	0.8219
TG	−0.1781	0.0207	−8.5905	<0.01	−0.2187	−0.1374
Int_1	0.0283	0.0094	3.0144	<0.01	0.0099	0.0467
Int_2	−0.0033	0.0018	−1.8893	0.0590	−0.0068	0.0001
Int_3	0.1267	0.0425	2.9805	<0.01	0.0433	0.2100
Int_4	−0.0110	0.0038	−2.8587	<0.01	−0.0185	−0.0034

*Note*: Int 1: ALAN × exercise; Int 2: ALAN × TG; Int 3: exercise × TG; Int 4: ALAN × exercise × TG.

Abbreviations: ALAN, artificial light at night; CVH, cardiovascular health; LLCI, lower‐level confidence interval; SE, standard error; TG: triglyceride; ULCI, upper‐level confidence interval.

**TABLE 10:** Moderation analysis between ALAN, exercise, TyG and CVH.

Variable	CVH					
	Coeff	SE	*t*	*p*	LLCI	ULCI
ALAN	0.0898	0.0518	1.7315	0.0835	−0.0119	0.1914
Exercise	−2.6148	1.0894	−2.4003	<0.05	−4.7514	−0.4782
TyG	−1.0203	0.0707	−14.4381	<0.01	−1.1589	−0.8817
Int_1	0.2243	0.0796	2.8193	<0.01	0.0683	0.3804
Int_2	−0.0078	0.0055	−1.4143	>0.05	−0.0186	0.0030
Int_3	0.3638	0.167	3.1166	<0.01	0.1349	0.5928
Int_4	−0.0228	0.0085	−2.7017	<0.01	−0.0394	−0.0063

*Note*: Int 1: ALAN × exercise; Int 2: ALAN × TyG; Int 3: exercise × TyG; Int 4: ALAN × exercise × TyG.

Abbreviations: ALAN, artificial light at night; CVH, cardiovascular health; LLCI, lower‐level confidence interval; SE, standard error; TyG: triglyceride glucose; ULCI, upper‐level confidence interval.

**TABLE 11:** Moderation analysis between ALAN, exercise, TG:HDL, and CVH.

Variable	CVH					
	Coeff	SE	*t*	*p*	LLCI	ULCI
ALAN	0.0266	0.0050	5.3257	<0.01	0.0168	0.0365
Exercise	0.6621	0.1013	6.5358	<0.01	0.4634	0.8608
TG‐HDL	−0.0586	0.0077	−7.6185	<0.01	−0.0737	−0.0435
Int_1	0.0222	0.0076	2.9210	<0.01	0.0073	0.0371
Int_2	−0.0005	0.0005	−0.9682	>0.05	−0.0015	0.0005
Int_3	0.0508	0.0139	3.6629	<0.01	0.0236	0.780
Int_4	−0.0046	0.0015	−3.1270	<0.01	−0.0075	−0.017

*Note*: Int 1: ALAN × exercise; Int 2: ALAN × TG‐HDL; Int 3: exercise × TG‐HDL; Int 4: ALAN × exercise × TG‐HDL.

Abbreviations: ALAN, artificial light at night; CVH, cardiovascular health; HDL, high‐density lipoprotein; LLCI, lower‐level confidence interval; SE, standard error; TG, triglyceride; ULCI, upper‐level confidence interval.

**TABLE 12:** Moderation analysis between ALAN, exercise, Alb, and CVH.

Variable	CVH					
	Coeff	SE	*t*	*p*	LLCI	ULCI
ALAN	0.0277	0.0047	5.8721	0.0743	0.0184	0.0369
Exercise	0.8847	0.0962	9.1949	<0.01	0.6959	1.0734
Alb	0.0014	0.0001	−4.6431	<0.01	−0.0007	−0.0003
Int_1	−0.0005	0.0063	0.8174	>0.05	−0.0108	0.0137
Int_2	0.00	0.000	−2.0873	<0.05	0.000	0.000
Int_3	−0.0002	0.0002	−1.2229	>0.05	−0.0006	0.0001
Int_4	0.000	0.000	2.2417	<0.05	0.000	0.0001

*Note*: Int 1: ALAN × exercise; Int 2: ALAN × Alb; Int 3: exercise × Alb; Int 4: ALAN × exercise × Alb.

Abbreviations: ALAN, artificial light at night; Alb, albumin; CVH, cardiovascular health; LLCI, lower‐level confidence interval; SE, standard error; ULCI, upper‐level confidence interval.

**TABLE 13:** Moderation analysis between ALAN, exercise, VSI, and CVH.

Variable	CVH					
	Coeff	SE	*t*	*p*	LLCI	ULCI
ALAN	0.0259	0.0051	5.1028	<0.01	0.0160	0.0359
Exercise	0.6278	0.1035	6.0666	<0.01	0.4248	0.8308
VSI	−0.0859	0.0125	−6.8477	<0.01	−0.1105	−0.0613
Int_1	0.0237	0.0078	3.0262	<0.01	0.0083	0.0391
Int_2	−0.0004	0.0008	−0.5644	>0.05	−0.0015	−0.0011
Int_3	0.0819	0.0212	3.8571	<0.01	0.0403	0.1236
Int_4	−0.0067	0.0021	−3.2122	<0.01	−0.0109	−0.0026

*Note*: Int 1: ALAN × exercise; Int 2: ALAN × VSI; Int 3: exercise × VSI; Int 4: ALAN × exercise × VSI.

Abbreviations: ALAN, artificial light at night; CVH, cardiovascular health; LLCI, lower‐level confidence interval; SE, standard error; ULCI, upper‐level confidence interval.

**TABLE 14:** Moderation analysis between ALAN, exercise, TyG‐BMI, and CVH.

Variable	CVH					
	Coeff	SE	*t*	*p*	LLCI	ULCI
ALAN	0.0383	0.0215	1.7857	0.0743	−0.0038	0.0804
Exercise	0.3568	0.5297	0.6735	>0.05	−0.6822	1.3958
TyG‐BMI	−0.0213	0.0012	−18.3987	<0.01	−0.0236	−0.0190
Int_1	0.0871	0.0337	2.5817	<0.01	0.0209	0.1532
Int_2	−0.0001	0.0001	−0.8493	>0.05	−0.0002	0.001
Int_3	0.0017	0.0021	0.8057	>0.05	−0.0025	0.0059
Int_4	−0.0003	0.0001	−2.3863	<0.05	−0.0006	−0.0001

*Note*: Int 1: ALAN × exercise; Int 2: ALAN × TyG‐BMI; Int 3: exercise × TyG‐BMI; Int 4: ALAN × exercise × TyG‐BMI.

Abbreviations: ALAN, artificial light at night; BMI, body mass index; CVH, cardiovascular health; LLCI, lower‐level confidence interval; SE, standard error; TyG, triglyceride glucose; ULCI, upper‐level confidence interval.

### 3.6 Moderation analysis between ALAN, NDVI, and metabolic metrics on CVH

Moderation analyses were performed for ALAN, NDVI, a metabolic metric (TG), and CVH. Among CVH, our results suggested a positive association between ALAN exposure and CVH; NDVI and TG play moderating roles in the increased risk of CVH induced by ALAN (Table 15). We observed similar results for ALAN, NDVI, vegetable intake, and CVH (Table 16).

**TABLE 15:** Moderation analysis between ALAN, NDVI, TG, and CVH.

Variable	CVH					
	Coeff	SE	*t*	*p*	LLCI	ULCI
ALAN	−0.1954	0.0653	−2.9939	<0.01	−0.3234	−0.0674
NDVI	−2.8062	1.0609	−2.6453	<0.01	−4.8869	−0.7256
TG	−0.3961	0.1500	−2.6417	<0.01	−0.6902	−0.1020
Int_1	0.6323	0.1722	3.5676	<0.01	0.2847	0.9799
Int_2	0.0429	0.0248	1.7279	0.0842	−0.0058	0.0916
Int_3	0.5122	0.2819	1.8168	0.0694	−0.0408	1.0651
Int_4	−0.1280	0.0678	−1.8865	0.0594	−0.2610	0.0051

*Note*: Int 1: ALAN × NDVI; Int 2: ALAN × TG; Int 3: NDVI × TG; Int 4: ALAN × NDVI × TG.

Abbreviations: ALAN, artificial light at night; CVH, cardiovascular health; NDVI, normalized difference vegetation index; LLCI, lower‐level confidence interval; SE, standard error; TG, triglyceride; ULCI, upper‐level confidence interval.

**TABLE 16:** Moderation analysis between ALAN, NDVI, vegetables, and CVH.

Variable	CVH					
	Coeff	SE	*t*	*p*	LLCI	ULCI
ALAN	−0.2059	0.0546	−3.7701	<0.01	−0.3131	−0.0988
NDVI	−1.1868	1.0420	−1.1389	>0.05	−3.2305	0.8570
Vegetable intake	0.2441	0.1388	1.7584	<0.01	−0.0282	0.5163
Int_1	0.6210	0.0546	4.2954	<0.01	0.3374	0.9045
Int_2	0.0177	0.0088	2.0047	<0.05	0.0004	0.0349
Int_3	−0.3262	0.2760	−1.1819	>0.05	−0.8676	0.2151
Int_4	−0.0483	0.0220	−2.1968	<0.05	−0.0914	−0.0052

*Note*: Int 1: ALAN × NDVI; Int 2: ALAN × vegetable; Int 3: NDVI × vegetable; Int 4: ALAN × NDVI × vegetable.

Abbreviations: ALAN, artificial light at night; CVH, cardiovascular health; LLCI, lower‐level confidence interval; NDVI, normalized difference vegetation index; SE, standard error; ULCI, upper‐level confidence interval.

### 3.7 Moderation analysis between ALAN, exercise, and CVH

Moderation analyses were performed for ALAN, drinking, walking, and CVH. The results are reported in Table S1. Among CVH, drinking and walking played moderating roles in the risk increase induced by ALAN. We observed similar results between ALAN, exercise, SES, and CVH (see Table S2).

### 3.8 Moderation analysis between ALAN, sleep duration, walking, and CVH

Moderation analyses were performed for ALAN, sleep duration, walking, and CVH (Table S3). Among CVH, sleep duration and walking played moderating roles in the risk increase of CVH induced by ALAN. We observed similar results in ALAN, sleep duration, sedentary behavior, and CVH (Table S4) as well as between ALAN, sleep duration, residential area, and CVH (Table S5).

### 3.9 Mediated moderation analysis between ALAN, sex, and CVH on CVD

Mediated moderation analyses were performed for ALAN, gender, CVH and CVD. The results are reported in Tables S6 and S7. Among CVD, gender plays a moderating role in the risk increase of CVD induced by ALAN mediated by CVH.

## 4 DISCUSSION

### 4.1 Principal results

This epidemiological survey examining diabetes among residents in four cities within Anhui Province established a positive correlation between exposure to residential outdoor ALAN and CVH. After adjusting for several significant diabetes‐related risk factors, these associations remained robust. We identified certain modifiable factors that moderated the impact of ALAN on CVH among individuals with diabetes. Our results build upon previous research^1^ and have important implications for evaluating the public health impact of light pollution on chronic diseases in China.

### 4.2 Comparison with previous research

Exposure to outdoor ALAN is a recognized risk factor for adverse health outcomes, including CVD.^36^ A recent animal study suggests that short‐term exposure to a common circadian disruptor, such as ALAN, increases inflammation and induces alterations in the angiogenic transcript within the mouse hippocampus.^37^ Night shift work can significantly affect circadian rhythms owing to exposure to nighttime light, electronic devices, and other factors. Individuals working night shifts face a heightened risk of developing metabolic disorders and several types of cancer. This also reflects the risk of exposure to nighttime light in other population segments. Individuals exposed to ALAN, including during mealtimes, also exhibit disrupted circadian rhythms, increased metabolism, and heart disease. External factors that disrupt circadian rhythms can impair cellular functions and insulin sensitivity, leading to impaired glucose tolerance and lipid metabolism rhythms. Thus, disrupted circadian rhythms can have considerable detrimental effects on glucose and lipid metabolism, potentially increasing the risk of impaired glucose tolerance and the transition to diabetes and abnormal lipid metabolism.^38^

Sleep is a vital biological process that can affect multiple functions, including cognition, development, energy conservation, and immune regulation.^39^ Some research supports the strong association between ALAN and sleep as well as its harmful effects on sleep. ALAN is closely associated with sleep‐related variables as it can disrupt the complete sleep cycle. The National China Child Health Study indicates that higher outdoor ALAN exposure levels are associated with a higher risk of sleep problems in children and are also more common in children under 12. This further suggests that effective control of outdoor ALAN exposure may be one of the key measures to improve children's sleep quality.^40^ A cross‐sectional study including older Chinese adults determined that an increase in nighttime light intensity located around the home outside was associated with the resulting decrease in sleep time.^41^ A cross‐sectional and longitudinal study of more than 400 000 UK Biobank participants also clarified that exposure to daytime light, even at low levels, is an important environmental risk factor that affects mood, sleep, and circadian rhythm‐related outcomes.^42^ A previous review highlighted the importance of causal relationships between outdoor ALAN, health, and behavior, and the importance of sleep as a key mediating factor,^12^ thus suggesting that sleep can mediate complex relationships between variables. This also suggests that keeping bedrooms dark and reducing exposure to outdoor light may be feasible measures to reduce the prevalence of sleep problems.^43^ A study comparing objective measures and self‐reports documented that reported sleep deprivation was significantly associated with cardiometabolic risk factors for T2DM. This highlights the need for vigilance to reduce the adverse impacts of sleep deprivation and several other factors.^44^

To further explore the role of HRBs in the development of CVH, a cross‐sectional study of the relationship between light exposure, sleep patterns, physical activity, and changes in melatonin levels in 61 female shift nurses highlighted the importance of melatonin levels.^45^ However, it did not establish a clear link between these variables, but determined that a consistent relationship between physical activity and melatonin, and sleep duration was not associated with urinary melatonin levels. These findings are particularly relevant because modifiable risk factors, such as changes in dietary and exercise patterns (low‐ to high‐intensity exercise), are often the primary targets for addressing cardiometabolic problems.^46^ A study examining the impact of shift work on CVD risk factors and MetS found that, even after accounting for potential covariates, such as job stress and dietary distribution, shift work was significantly associated with MetS.^47^ This highlights the need to consider the metabolic effects of ALAN while also acknowledging the potential moderating roles of physical activity and sedentary behaviors.^1^ Other studies suggest that physical activity is inversely associated with the incidence and progression of cardiometabolic diseases.^48^, ^49^ A prospective UK Biobank study revealed that long‐term exposure to PM2.5 and NO_2_ are linked to the prognosis of cardiometabolic multimorbidity. Older participants, men, individuals who drink excessive alcohol, and those with lower SES are more likely to experience these risks.^50^ The US Center for Disease Control and Prevention reported a correlation between the Social Vulnerability Index and ALAN.^51^ To further clarify the interaction between ALAN and CVH in CVD, we conducted corresponding analyses, thus providing supplementary evidence to support the importance of considering the association between ALAN and CVD. Previous studies document that participants who live in areas with higher grass and tree cover have lower rates of MetS associated with ALAN,^21^ which is consistent with our results. This suggests the importance of developing green spaces, particularly in areas experiencing active or passive increases in ALAN.

The associated mechanisms include a direct effect of ALAN on the endocrine rhythms of the central nervous system, including the hypothalamic–pituitary–adrenal (HPA) axis and the hormone feedback mechanism that regulates its activity.^52^ From a possible mechanism viewpoint, ALAN is absorbed by various circulatory systems through the brain. Furthermore, hormones such as corticotropin releasing and arginine antidiuretic hormones are involved in the daily release of corticosterone and regulated by clock genes.^53^ Melatonin is mainly involved in regulating the circadian cycle in the body, and its production and secretion are partly regulated by external light signals; therefore, it will also be affected by ALAN^54^ and thus affect the secretion. Some studies indicate that the disruption of melatonin concentration is related to the changes of HPA axis rhythm,^55^, ^56^ thereby suggesting that ALAN exposure and the disruption of melatonin rhythm may further exacerbate neuroendocrine and metabolic disorders, resulting in a series of metabolic diseases. Fleury et al. reviewed animal experiments and population studies and revealed that exposure to ALAN causes circadian rhythm disruption, which negatively affects metabolic health.^57^ Another perspective is the relationship between nighttime light exposure and altered metabolic function through changes in sleep. Since light exposure affects sleep, alterations in sleep patterns may be a primary mechanism accounting for the changes in glucose regulation.^36^ ALAN disrupts the circadian rhythm to some extent by inhibiting the secretion of melatonin at night, and changes daily behavior such as exercise, sleep, and diet, which may lead to the development of metabolic diseases.^58^ Detailed mechanisms between ALAN and circadian rhythms regarding cardiovascular and metabolic diseases including AMPK‐OPA1‐mitochondrial fusion/mitophagy axis, and circadian‐related genes. Furthermore, the circadian clock plays a key role in linking obesity to the clock mechanisms of fat cells.^59^ Another mechanism includes the association between ALAN and the inflammatory response, particularly in relation to the rhythm of inflammatory factors.^60^, ^61^ Animal experiments established that the expression of metabolic transcription factors Pparα and Pparγ increased in the epididymal fat of the low‐dose ALAN group and the expression of Glut4 decreased in the heart.^62^ In addition to these harmful factors, the role of protective factors in CVH is also significant, including green spaces and healthy behaviors. These are viewed as potential mechanisms linking human health with the “active component” in nature. They have been recognized as having some degree of influence on health, including physical/mental states, behaviors, and conditions related to nature and health, including intense sleep and immune responses; specific health outcomes associated with nature (controlling for socioeconomic variables), including CVD,^63^, ^64^ are associated to some extent.

### 4.3 Limitations and strengths

The strengths of this study include results that align with those reported previously, specifically the interaction between ALAN and various behavioral patterns with CVH in patients with diabetes. Furthermore, we have developed corresponding countermeasures, thus providing a sound theoretical basis for further research focused on reducing ALAN exposure and effectively preventing the development of CVH through healthy behavioral interventions. Additional strengths include the multi‐factor and multilevel study design, as well as the large number of participants, which facilitates comprehensive coverage of ALAN exposure levels across different cities.

Conversely, several limitations must be noted. First, the variables were all self‐reported, and the imprecision and potential bias of their measurement associations limit our ability to further assess causality accurately. Second, our observational study faces challenges in establishing a causal relationship between ALAN, HRBs, and CVH. As an observational study, causation cannot be inferred. Thus, further cohort studies should be conducted to confirm and strengthen the potential association between outdoor ALAN exposure and disease risk across various groups. Third, we focused on such exposure without considering the effect of indoor lighting, particularly nighttime light exposure during sleep, on cardiometabolic outcomes. Finally, this study is limited to some cities in Anhui Province. Follow‐up studies will be conducted with participants from different regions and cultures across the country. Since early detection and intervention are particularly effective in the treatment of future complications, this will include younger patients in particular. Future research will include additional mechanisms of circadian rhythm‐related variables contributing to cardiac metabolic disease, prospective studies, and clinical trials.

## 5 CONCLUSIONS

We explored the relationship between ALAN and behaviors, metabolic metrics, and CVH. The results revealed a high prevalence rate of behavior patterns and metabolic metrics in patients with T2DM in Anhui Province. Circadian rhythms can be disrupted by environmental and behavioral factors, such as ALAN, sleep duration, and sedentary behavior. By exploring the environmental factors that influence diabetes and CVD, especially light pollution, our findings can promote the effective treatment of light pollution and help design strategies to combat public health challenges.

## AUTHOR CONTRIBUTIONS

QZ and YZ constructed the study design. QZ recruited the participants. YZ, YT, KYH, CL, and TJ were involved in statistical analysis. QZ, FBT, and HG were responsible for the critical revision of the manuscript. YZ, QF, XC, CHS, CH, and LWC edited and revised the manuscript. YZ, YT, and KYH prepared and drafted the manuscript. All the authors who contributed to the manuscript gave their approval for its submission. The work presented here has not been published previously and is not being considered for publication elsewhere. All authors have read and approved the manuscript.

## FUNDING INFORMATION

The author(s) disclose receipt of the following financial support for the research, authorship, and/or publication of this article: Funding for the project was provided by China Postdoctoral Science Foundation (2023M740022), Research Fund of Anhui Institute of translational medicine (2023 hyx‐C33), and National Natural Science Foundation of China (82370836 and 81970703).

## CONFLICT OF INTEREST STATEMENT

The authors declare no conflicts of interest.

## Supporting information


**Table S1.** The moderation analysis between ALAN, walking, drinking and CVH.
**Table S2.** The moderation analysis between ALAN, walking, SES and CVH.
**Table S3.** The moderation analysis between ALAN, walking, sleep duration and CVH.
**Table S4.** The moderation analysis between ALAN, SB, sleep duration and CVH.
**Table S5.** The moderation analysis between ALAN, residential areas, sleep duration and CVH.
**Table S6.** Model characteristics for the conditional process analysis.
**Table S7.** Bootstrapped conditional direct and indirect effects.
