# Supplementary material for: Interactive correlations between artificial light at night, health risk behaviors, and cardiovascular health among patients with diabetes: A cross‐sectional study
Source: J Diabetes. 2024 Oct 13;16(10):e70008. doi: 10.1111/1753-0407.70008 (PMC11471435; doi:10.1111/1753-0407.70008)
Supplement: Supplementary file 1 — Table S1. The moderation analysis between ALAN, walking, drinking and CVH. Table S2. The moderation analysis between ALAN, walking, SES and CVH. Table S3. The moderation analysis between ALAN, walking, sleep duration and CVH. Table S4. The moderation analysis between ALAN, SB, sleep duration and CVH. Table S5. The moderation analysis between ALAN, residential areas, sleep duration and CVH. Table S6. Model characteristics for the conditional process analysis. Table S7. Bootstrapped conditional direct and indirect effects. [file JDB-16-e70008-s001.docx]

| Table S1 The moderation analysis between ALAN, walking, drinking and CVH | | | | | | |
| --- | --- | --- | --- | --- | --- | --- |
| Variables | CVH | | | | | |
|  | coeff | se | t | P | LLCI | ULCI |
| ALAN | -0.0042 | 0.0361 | -0.1154 | ＞0.05 | -0.0750 | 0.0666 |
| Walking | -0.0029 | 0.3409 | -0.0084 | ＞0.05 | -0.6714 | 0.6657 |
| Drinking | -0.4973 | 0.3821 | -1.3018 | ＞0.05 | -1.2467 | 0.2520 |
| Int_1 | 0.0364 | 0.0253 | 1.4369 | ＞0.05 | -0.0133 | 0.0861 |
| Int_2 | 0.0449 | 0.0256 | 1.7497 | 0.0803 | -0.0054 | 0.0952 |
| Int_3 | -0.1684 | 0.2447 | -0.6883 | ＞0.05 | -0.6484 | 0.3115 |
| Int_4 | -0.0419 | 0.0179 | -2.3368 | ＜0.05 | -0.0771 | -0.0067 |
| Int 1: ALAN × walking; Int 2: ALAN × drinking; Int 3: walking × drinking; Int 4: ALAN × walking × drinking. | | | | | | |

| Table S2 The moderation analysis between ALAN, walking, SES and CVH | | | | | | |
| --- | --- | --- | --- | --- | --- | --- |
| Variables | CVH | | | | | |
|  | coeff | se | t | P | LLCI | ULCI |
| ALAN | 0.1219 | 0.0407 | 2.9913 | ＜0.01 | 0.0420 | 0.2017 |
| Walking | -0.0412 | 0.2639 | -0.1562 | ＞0.05 | -0.5587 | 0.4763 |
| SES | 0.3386 | 0.2165 | 1.5638 | ＞0.05 | -0.0861 | 0.7633 |
| Int_1 | -0.0658 | 0.0273 | -2.4127 | ＜0.05 | -0.1193 | -0.0123 |
| Int_2 | -0.0343 | 0.0184 | -1.8619 | 0.0628 | -0.0705 | 0.0018 |
| Int_3 | -0.0902 | 0.1386 | -0.6506 | ＞0.05 | -0.3620 | 0.1817 |
| Int_4 | 0.0230 | 0.0126 | 1.8324 | 0.0671 | -0.0016 | 0.0477 |
| Int 1: ALAN × walking; Int 2: ALAN × SES; Int 3: walking × SES; Int 4: ALAN × walking × SES. | | | | | | |

| Table S3 The moderation analysis between ALAN, walking, sleep duration and CVH | | | | | | |
| --- | --- | --- | --- | --- | --- | --- |
| Variables | CVH | | | | | |
|  | coeff | se | t | P | LLCI | ULCI |
| ALAN | -0.0259 | 0.0421 | -0.6160 | ＞0.05 | -0.1085 | 0.0566 |
| Walking | -0.7161 | 0.4138 | -1.7307 | 0.0837 | -1.5277 | 0.0957 |
| Sleep duration | -0.4956 | 0.2708 | -1.8302 | ＞0.05 | -1.0267 | 0.0355 |
| Int_1 | 0.0411 | 0.0296 | 1.3874 | ＞0.05 | -0.0170 | 0.0992 |
| Int_2 | 0.0356 | 0.0184 | 1.9319 | 0.0535 | -0.0005 | 0.0717 |
| Int_3 | 0.2219 | 0.1751 | 1.2672 | ＞0.05 | -0.1216 | 0.5654 |
| Int_4 | -0.0268 | 0.0129 | -2.0816 | ＜0.05 | -0.0521 | -0.0016 |
| Int 1: ALAN × walking; Int 2: ALAN × sleep duration; Int 3: walking × sleep duration; Int 4: ALAN × walking × sleep duration. | | | | | | |

| Table S4 The moderation analysis between ALAN, SB, sleep duration and CVH | | | | | | |
| --- | --- | --- | --- | --- | --- | --- |
| Variables | CVH | | | | | |
|  | coeff | se | t | P | LLCI | ULCI |
| ALAN | 0.0712 | 0.0333 | 2.1348 | ＜0.05 | 0.0058 | 0.1365 |
| SB | 0.0227 | 0.0915 | 0.2482 | ＞0.05 | -0.1567 | 0.2021 |
| Sleep duration | -0.0948 | 0.0549 | -1.7278 | 0..0842 | -0.2025 | 0.0128 |
| Int_1 | -0.0147 | 0.0058 | -2.5360 | ＞0.05 | -0.0260 | -0.0033 |
| Int_2 | -0.0034 | 0.0049 | -0.7076 | ＞0.05 | -0.0130 | 0.0061 |
| Int_3 | -0.0022 | 0.0126 | -0.1782 | ＞0.05 | -0.0270 | 0.0225 |
| Int_4 | 0.0018 | 0.0009 | 2.0548 | ＜0.05 | 0.0001 | 0.0035 |
| Int 1: ALAN × walking; Int 2: ALAN × sleep duration; Int 3: walking × sleep duration; Int 4: ALAN × walking × sleep duration. | | | | | | |

| Table S5 The moderation analysis between ALAN, residential areas, sleep duration and CVH | | | | | | |
| --- | --- | --- | --- | --- | --- | --- |
| Variables | CVH | | | | | |
|  | coeff | se | t | P | LLCI | ULCI |
| ALAN | -0.1107 | 0.0433 | -2.5534 | ＜0.05 | -0.1957 | -0.0257 |
| Sleep duration | -0.2108 | 0.1469 | -1.4349 | ＞0.05 | -0.4990 | 0.0773 |
| Residential areas | -0.7437 | 0.3857 | -19280 | 0.0540 | -1.5003 | 0.0129 |
| Int_1 | 0.0169 | 0.0063 | 2.6618 | ＜0.01 | 0.0044 | 0.0293 |
| Int_2 | 0.0540 | 0.0210 | 2.5657 | ＜0.05 | 0.0127 | 0.0953 |
| Int_3 | 0.0437 | 0.0546 | 0.8013 | ＞0.05 | -0.0633 | 0.1508 |
| Int_4 | -0.0062 | 0.0031 | -1.9813 | ＜0.05 | -0.0124 | -0.0001 |
| Int 1: ALAN × sleep duration; Int 2: ALAN × residential; Int 3: residential × sleep duration; Int 4: ALAN × residential × sleep duration. | | | | | | |

| Table S6 Model characteristics for the conditional process analysis. | | | | | | |
| --- | --- | --- | --- | --- | --- | --- |
| Variables | CVH | | | CVD | | |
|  | B | t value | *P* value | B | t value | *P* value |
| ALAN | 0.0551 | 4.3963 | ＜0.01 | 0.0073 | 2.0259 | ＜0.05 |
| Gender | 0.9749 | 8.6420 | ＜0.01 | -0.4934 | -2.8255 | ＜0.01 |
| ALAN*gender | -0.0129 | -1.6282 | ＞0.05 | -0.0049 | -2.1680 | ＜0.01 |
| CVH |  |  |  | -0.0513 | -2.4700 | ＜0.05 |
| CVH*gender |  |  |  | 0.0397 | 2.9977 | ＜0.01 |
| R^2^ | 0.0892 | | | 0.0078 | | |
| F | 57.1313 | | | 27.55 | | |
| Mediate variables: CVH, moderated variables: gender, independent variables: ALAN, dependent variables: CVD. uncontrolled | | | | | | |

| Table S7 Bootstrapped conditional direct and indirect effects. | | | | | |
| --- | --- | --- | --- | --- | --- |
|  |  | | CVD | | |
| Direct effect |  |  | Effect | SE | (LL,UL) |
|  | Predictor | CVH |  |  |  |
|  | Moderator | Male | 0.0025 | 0.0016 | -0.0007,0.0057 |
|  |  | Female | -0.0024 | 0.0015 | -0.0054,0.0006 |
| Indirect effect |  |  | Effect | SE | (LL,UL) |
|  | Predictor | Resilience |  |  |  |
|  | Mediator | Low | -0.0005 | 0.0004 | -0.0013,0.0002 |
|  |  | High | 0.0006 | 0.0002 | 0.0003,0.0012 |
